# Bach Is the Father of Harmony: Revealed by a 1/f Fluctuation Analysis across Musical Genres

**DOI:** 10.1371/journal.pone.0142431

**Published:** 2015-11-06

**Authors:** Dan Wu, Keith M. Kendrick, Daniel J. Levitin, Chaoyi Li, Dezhong Yao

**Affiliations:** 1 Department of Biomedical Engineering, School of Computer and Information Technology, Beijing Jiaotong University, Beijing, China; 2 Key Laboratory for NeuroInformation of Ministry of Education, School of Life Science and Technology, University of Electronic Science and Technology of China, Chengdu, China; 3 Department of Psychology, McGill University, Montreal, Canada; 4 Center for Life Sciences, Shanghai Institutes for Biological Sciences, Chinese Academy of Sciences, Shanghai, China; University of Pécs Medical School, HUNGARY

## Abstract

Harmony is a fundamental attribute of music. Close connections exist between music and mathematics since both pursue harmony and unity. In music, the consonance of notes played simultaneously partly determines our perception of harmony; associates with aesthetic responses; and influences the emotion expression. The consonance could be considered as a window to understand and analyze harmony. Here for the first time we used a 1/f fluctuation analysis to investigate whether the consonance fluctuation structure in music with a wide range of composers and genres followed the scale free pattern that has been found for pitch, melody, rhythm, human body movements, brain activity, natural images and geographical features. We then used a network graph approach to investigate which composers were the most influential both within and across genres. Our results showed that patterns of consonance in music did follow scale-free characteristics, suggesting that this feature is a universally evolved one in both music and the living world. Furthermore, our network analysis revealed that Bach’s harmony patterns were having the most influence on those used by other composers, followed closely by Mozart.

## Introduction

Throughout history, music has played an important role in people’s daily lives [1]. Many studies have attempted to discover why music can so powerfully influence our mood [2–5]. Mathematics and physics have often been used to characterize, analyze, model and understand music. For example, the musical chords and voice leadings were modelled by geometric space [6,7]. The topology analysis [8] and compositional data analysis [9] were both used to investigate musical structures. Johannes Kepler’s “*The Harmony of the World*” was inspired by music [10]. Musical elements and structures have been found to follow a 1/f distribution, termed “fractal” by Mandelbrot [11]. The power spectra of musical signals decays in a power law with frequency f as [1/f]^β^ (where β is the spectral exponent). The DFA (detrended fluctuation analysis) exponent (also called Hurst exponent) is denoted as α, and it is related to β via α = (β+1)/2. Hence, a DFA exponent α may be translated to an asymptotic scaling exponent β = 2*α-1, and the power spectral density can be represented as p(f)~[1/f]^2*α-1^. In this work, the DFA exponent α is referred to as the scaling exponent. A signal is considered long-range correlated if its power spectral density (PSD) asymptotically decays in a power law, p(f)∼[1/f]^β^ for small frequencies f and 0<β<2 [12]. The limit β = 0 indicates white noise, the structure of which is entirely unpredictable [13]; β = 2 is Brownian motion. Hence, long-range correlations are found in a signal when 0.5<α<1.5. When α = β = 1, the signal is the 1/f noise.

The aesthetics of music has been shown to be related to its scale-free exponent [14] when applied to pitch structures. Much of the enjoyment of music relates to the balance of predictability and surprise [13]. The 1/f distribution probably indicates such balance. Indeed, many specific musical elements such as pitch, melody and rhythm follow the scale-free law [13,15–17]. The 1/f distribution of note pitch in music is supported by different hierarchies: the power spectral analysis of audio waves [14], the frequency of occurrence of all the notes [15], and the fluctuation of pitch [18]. For melody, the structure of self-similarity is expressed more directly [17]. The rhythm, which is considered highly regular and predictable, is also proved to obey the 1/f power law [13]. Musical performances also display 1/f properties in expressive tempo fluctuations, and listeners predict tempo changes when synchronizing [19]. And such preference in rhythm is observed in human perception and musical performance [12,20]. The fractal structure of songs can be influenced by performer's preference [21], while the synchronization and accuracy of human movements can also be effected by the consonance and dissonance of music [22]. In addition, 1/f noise in the timing of musical performance can be used to assess motoric dysfunctions [23]. These findings suggest that the scale-free characteristic of music is an important intrinsic property which may reflect not only a musician’s individual unique contribution but also the learned or acquired influence of previous composers and genres.

The harmony, consonance of notes played simultaneously, is an important feature in Western music. Traditional music theory describes the rules for the use of these harmony intervals or chords in composition [24]. In Western music, dissonance is the quality of sounds that are perceived as “unstable” and have an aural “need” to “resolve” to a “stable” consonance. There are many relative constant patterns of chord progressions. When one chord occurs, the next chord will be expected or predicted quite easily according to the patterns. Actually, the expectations of harmony can influence the emotion [25]. To describe the harmony rules, a geometric model is used, in which a chord can be represented as a point in a geometrical space and line segments represent mappings from the notes of one chord to those of another [7]. However, the fluctuations of the consonance in real musical pieces may change even in one beat if there is more than one note played consecutively in this beat, so the harmony variation may not be regular according to the theory.

The harmony, as a mark of a composer, can be used to identify the composers or genres [26,27]. Several studies used networks to describe the relationships among the composers. The network of notes in a score, and that of different musicians, has been evaluated based on the association between notes or their subjective similarity judged by musical editors [28]. Two composers are considered having close relationship when their works appeared in one record [29], on the same webpage [30], or in one playlist [31]. Most networks of composers are established by subjective judgment. In this study, we show that a network based on consonance fluctuations of the composers and genres can reveal some intrinsic properties of music.

Here we are interested in the intrinsic mutual relationships among typical musical pieces within and across different eras according to their mathematical characteristics. We try to find the characteristics of consonance fluctuations for different composers and genres, and networks of composers and genres are established according to similarity of musical harmony. Because music in the western tradition builds on the styles and structures of previously written music, compositions from different eras are not entirely independent from one another. Here we seek to quantify the latent structure underlying musical pieces through their harmony fluctuations spanning composers and genres. In general, harmonic fluctuation may be characterized by chord,since stability and predictability are the soul of chord progression in a work, we pay special attention to the stability of chord which are related to the interval consonance, so we use the fluctuation of pairwise consonance to approximate this property (S1 Table), such an approach is based on the basic physical properties of notes thus more direct and understandable.

## Materials and Methods

### Processing of the musical scores

We selected 1191 musical movements from 568 compositions written by 20 composers from across 9 different genres spanning from the late 16^th^ to the early 20^th^ century. As in prior work, each movement was treated as an independent piece [13]. MIDI files were obtained for analysis from the Humdrum Kern database [32], allowing the pitch, duration and onset time of all the notes to be automatically extracted. All the files can be found in the Supporting Information S1 File. At least 9 compositions with an average of 60 movements were analyzed for each composer, as summarized in Table 1. Nine different genres were selected and at least 13 movements were evaluated for each genre, as summarized in Table 2.

**Table 1:** Statistics for the compositions of different composers.

Composer (period)	Number of movements	α (original)	α (shuffled)	*P* value
Bach (1685–1750)	145	0.86 ± 0.09	0.51 ± 0.03	P<0.001
Beethoven (1770–1827)	161	0.86 ± 0.07	0.51 ± 0.03	P<0.001
Brahms (1833–1897)	9	0.86 ± 0.05	0.49 ± 0.04	P<0.001
Buxtehude (1639–1707)	20	0.92 ± 0.16	0.50 ± 0.04	P<0.001
Chopin (1810–1849)	84	0.87 ± 0.09	0.51 ± 0.04	P<0.001
Clementi (1756–1832)	17	0.79 ± 0.09	0.51 ± 0.04	P<0.001
Corelli (1653–1713)	130	0.94 ± 0.12	0.51 ± 0.04	P<0.001
Frescobaldi (1583–1643)	40	0.90 ± 0.06	0.50 ± 0.03	P<0.001
Grieg (1843–1907)	16	0.84 ± 0.09	0.52 ± 0.03	P<0.001
Haydn (1732–1809)	158	0.87 ± 0.08	0.50 ± 0.03	P<0.001
Hummel (1778–1837)	24	1.09 ± 0.13	0.54 ± 0.05	P<0.001
Joplin (1868–1917)	45	0.80 ± 0.08	0.52 ± 0.03	P<0.001
MacDowell (1860–1908)	9	0.91 ± 0.11	0.49 ± 0.03	P<0.001
Monteverdi (1567–1643)	12	0.92 ± 0.05	0.51 ± 0.03	P<0.001
Mozart (1756–1791)	160	0.82 ± 0.08	0.50 ± 0.03	P<0.001
Scarlatti (1685–1757)	59	0.79 ± 0.07	0.51 ± 0.03	P<0.001
Schubert (1797–1828)	21	0.80 ± 0.12	0.52 ± 0.03	P<0.001
Scriabin (1872–1915)	13	0.85 ± 0.12	0.52 ± 0.04	P<0.001
Sousa (1854–1932)	10	0.85 ± 0.10	0.51 ± 0.03	P<0.001
Vivaldi (1678–1741)	58	0.91 ± 0.11	0.51 ± 0.04	P<0.001

For a ± b, a is the mean value, and b is the standard deviation.

**Table 2:** Statistics for the compositions of different genres.

Genres	Number of movements	α (original)	α (shuffled)	*P* value
Etude	19	0.85 ± 0.11	0.51 ± 0.04	P<0.001
Fugue	62	0.88 ± 0.06	0.50 ± 0.03	P<0.001
Mazurka	52	0.88 ± 0.08	0.51 ± 0.03	P<0.001
Prelude	87	0.94 ± 0.13	0.51 ± 0.05	P<0.001
Quartet	305	0.87 ± 0.08	0.51 ± 0.03	P<0.001
Ragtime	21	0.78 ± 0.09	0.51 ± 0.03	P<0.001
Sonata	378	0.86 ± 0.12	0.51 ± 0.04	P<0.001
Sonatina	26	0.81 ± 0.09	0.50 ± 0.03	P<0.001
Waltz	13	0.85 ± 0.07	0.51 ± 0.03	P<0.001

For a ± b, a is the mean value, and b is the standard deviation.

**Fig 1:**
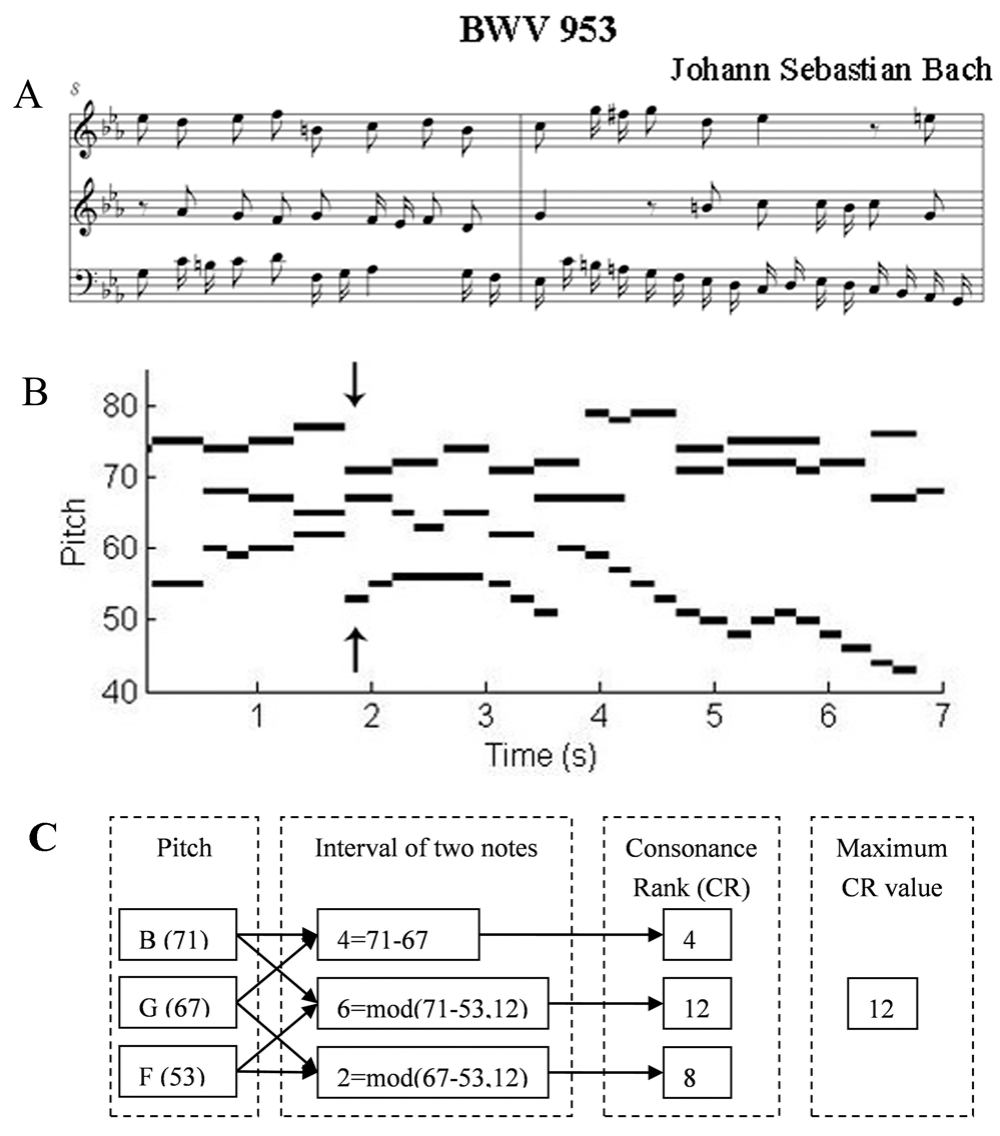
The extraction of the consonance rank series from a musical score. (A) An example musical score from Bach’s work BWV 953. (B) The MIDI information corresponding to the piece of music in A; the arrows represent the time point for the consonance rank (CR) series computation. (C) The steps for the CR series calculation. The pitches are initially obtained from the MIDI information (at the given arrow point, the pitches are B4 (71), G4 (67), F3 (53)); then, the intervals of every two notes are calculated, and the intervals that exceeded one octave (12 semitones) are adjusted to one octave. Subsequently, the intervals are translated to the CR according to the mapping rule in Table 3; finally, the maximum CR value is acquired as the value for the CR series at the given time point.

A musical score represents the pitch and duration of each note in a musical piece, as is shown with an example in Fig 1A. Here we first changed the score into a graph, with x axis as time and y axis as the pitch. In MIDI notation, each pitch corresponds to a number. For example, the middle C, C4 in scientific pitch notation (SPN), 261.63 Hz, is 60. Each note now can be represented by a line segment in the graph. The starting point represents the time when a note is on, and the end point represents the note off, as in Fig 1B. Then the consonance was computed, and a curve of consonance was obtained. The consonance of the musical intervals corresponds to the ratio of the frequency of the notes. The ratio 2:1 produces an octave; 3:2 produces a fifth and so on. There are many types of note combinations; some are consonant (e.g., the perfect fifth with a frequency ratio of 3:2), and some are dissonant (e.g., the minor second with a frequency ratio of 16:15). The corresponding consonant rank for all the intervals in an octave is shown in Table 3. To measure the consonance of intervals, roughness, an auditory attribute, was proposed by Helmholtz as a sensory basis for musical consonance within the tonal system [33]. Here we adopted the consonant rank (CR) as the measure, which was a visually direct, convenient but coarse-grained value of the roughness.

**Table 3:** The mapping rule for pitch interval to the consonance rank.

Intervals (semitone)	Interval name	Consonance rank
0 or 12	unison/octave	1
1	minor second	11
2	major second	8
3	minor third	6
4	major third	4
5	perfect fourth	3
6	augmented fourth/diminished fifth	12
7	perfect fifth	2
8	minor sixth	7
9	major sixth	5
10	minor seventh	9
11	major seventh	10

A musical harmony/chord progression is defined by a change from a “stable” condition to an “unstable” condition and then back to a “stable” condition. Here, a stable pitch combination often consists of consonant intervals, whereas the dissonant intervals often induce unstable feelings. However, as consonance and dissonance are relative in music, we do not discriminate them explicitly for each time point; we just calculated the note pitch intervals between every two notes first in Fig 1C. Intervals that exceeded one octave (12 semitones) were converted into one octave with the mode of 12. And based on the corresponding consonant rank (CR) (Table 3), the CR values of the intervals were found out. Then the maximum CR was taken to represent the relatively dissonant interval of this moment (Fig 1C). At last, we obtained a curve of the CR values for each music piece.

In this way, we pay more attention to the relative dissonant intervals. In fact, dissonance is not noise or redundancy in music; on the contrary, it plays a prominent role in many traditional musical cultures, even being considered to be the main motivation for musical progression. In short, a consonant interval is the main body and elemental requirement of a piece of almost acknowledged musical works, and it is the specific use of the relative dissonance that may lead to the differences in music genres and styles of musicians.

### Detrended fluctuation analysis

Detrended fluctuation analysis (DFA) is a useful tool for analyzing the nonlinear dynamic properties of a system [34]; it is also utilized to estimate the scaling exponent in a power law distribution [13]. Here the DFA is used to obtain the scaling exponent of the musical CR value curves. In detrended fluctuation analysis, the time series with number of N samples is integrated as.

$$y(m)=\sum_{t=1}^{m}\left[x(t)-\bar{x}\right]$$ (1)

where *x*(*t*) is the sequence at time *t*, and $\bar{x}$ is the average of the entire time series. Then *y*(*m*), integrated time series, is divided into subsequences of equal length L. In each window, the y-coordinate of a least-square line which fits to the data is denoted by *y*_*L*_(*m*). Finally, the average fluctuation as a function of window size L is given by.

$$F(L)=\sqrt{\frac{1}{N}\sum_{t=1}^{N}{\left[y(m)-y_{L}(m)\right]}^{2}}$$ (2)

If there is a straight line on a log-log graph, it signifies a statistical self-affinity expressed as *F*(*L*) ∝ *L* ^−*α*^. The scaling exponent α is calculated as the slope of a straight line fit to the log-log graph of *L* against *F(L)* using a least-squares regression. If the exponent is less than 0.5, the signal is anti-correlated; if the exponent is 0.5, the signal is uncorrelated (white noise). If the exponent is greater than 0.5, the signal may be correlated. An α value of 1 indicates 1/f noise, which is called scale free.

**Fig 2:**
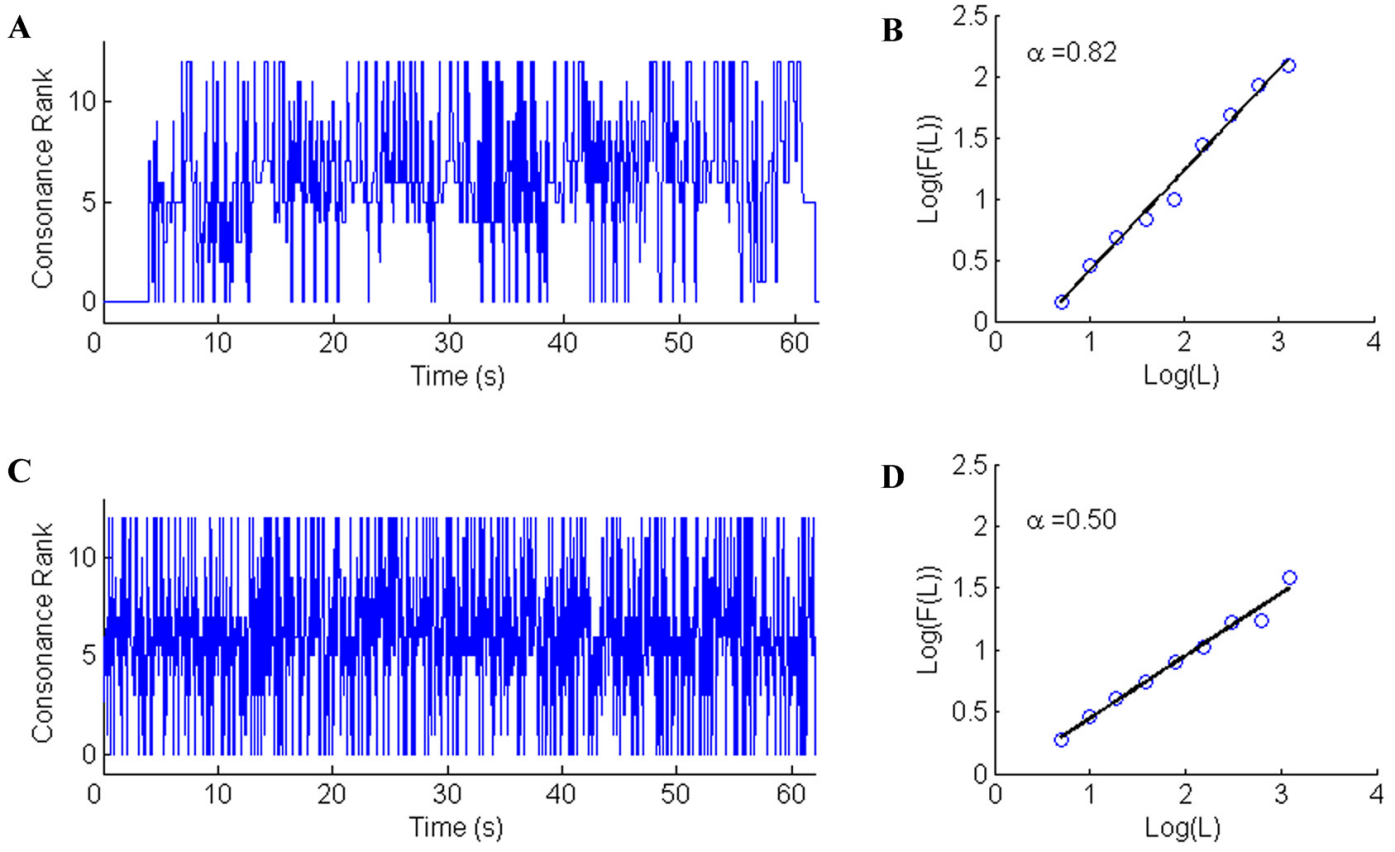
The consonance rank series and scaling exponent of BWV 953 and shuffled signal. (A) The consonance rank series of BWV 953. (B) The scaling exponent of the signal in A. (C) The shuffled series related to the signal in A. (D) The scaling exponent of the shuffled series.

The scaling exponent α was calculated from the CR time series and we calculated the scaling exponent for the CR series for each musical movement. The length of each musical movement was different, the average CR time series length was 3777 and the shortest was 600 (sampling rate was 20 Hz). Fig 2A showed the CR time series of the BWV 953. And Fig 2B was the scaling exponent of this music piece. For statistical comparison, a shuffled random signal was generated (an example was shown in Fig 2C) and the scaling exponent was computed (Fig 2D). For every musical movement, the CR series would be randomly shuffled so that each piece of music would have a contrastive signal. Thus the consonance of the musical movement was kept in a random sequence. The Wilcoxon signed rank test was used to test the difference between the CR series and the shuffled counterparts and statistical tests were performed in Matlab. A two-tailed t test was used to analyze the differences across the composers and genres.

### Networks of composers and genres

The established composer network was based on the differences among the composers. The genre network was based on the differences among the genres. The composers/genres were the nodes in the graph. When two musicians or two genres had no significant differences (t test, *P*>0.05), a connection was considered to exist between both. We focused on the degree, the out-in degree, and the modularity of the network.

In graph theory, the degree of a vertex (node) is the number of edges linked to the vertex (node). When it is a directed network, there are two kinds of edges for a node, the edges originating from the node (out) and those directed into it (in). The out-in-degree is the difference between the number of out-going lines and the number of incoming lines. In our composers’ network, the directions of the edges were defined according to the time they were born, thus the edge was from the composer who was born early to the one born late.

The “community structure” is useful for analyzing the relationship among the musicians. It divides them naturally into communities or modules with dense connections within communities but sparser connections between them. The modularity is a statistic that quantifies the degree to which the network may be subdivided into such clearly delineated groups [35]. In this study, we used a method to find the optimal community structure [36], which was a subdivision of the network into non-overlapping groups of nodes in a way that maximized the number of within-group edges but minimized the number of between-group edges. The result of community structure in this study was the average for 100 runs.

## Results

The scaling exponent (α) was calculated after the interval consonant rank (CR) series were extracted from a musical movement. The α of the exampled music (Bach's BWV953) is 0.82 (Fig 2B). This means that in the log-domain, the fluctuation increased exponentially with the window size of the sequence as 1/f. Fig 2D illustrates the scaling exponent of the shuffled signal of the example musical movement. The α is 0.5, which means that the shuffled signal is just white noise. This finding indicates that the 1/f fluctuation reflects the global structure across the entire piece, and this structure is a consequence of the specific ordering of the harmony sequence, not their mere presence in the piece at random locations.

**Fig 3:**
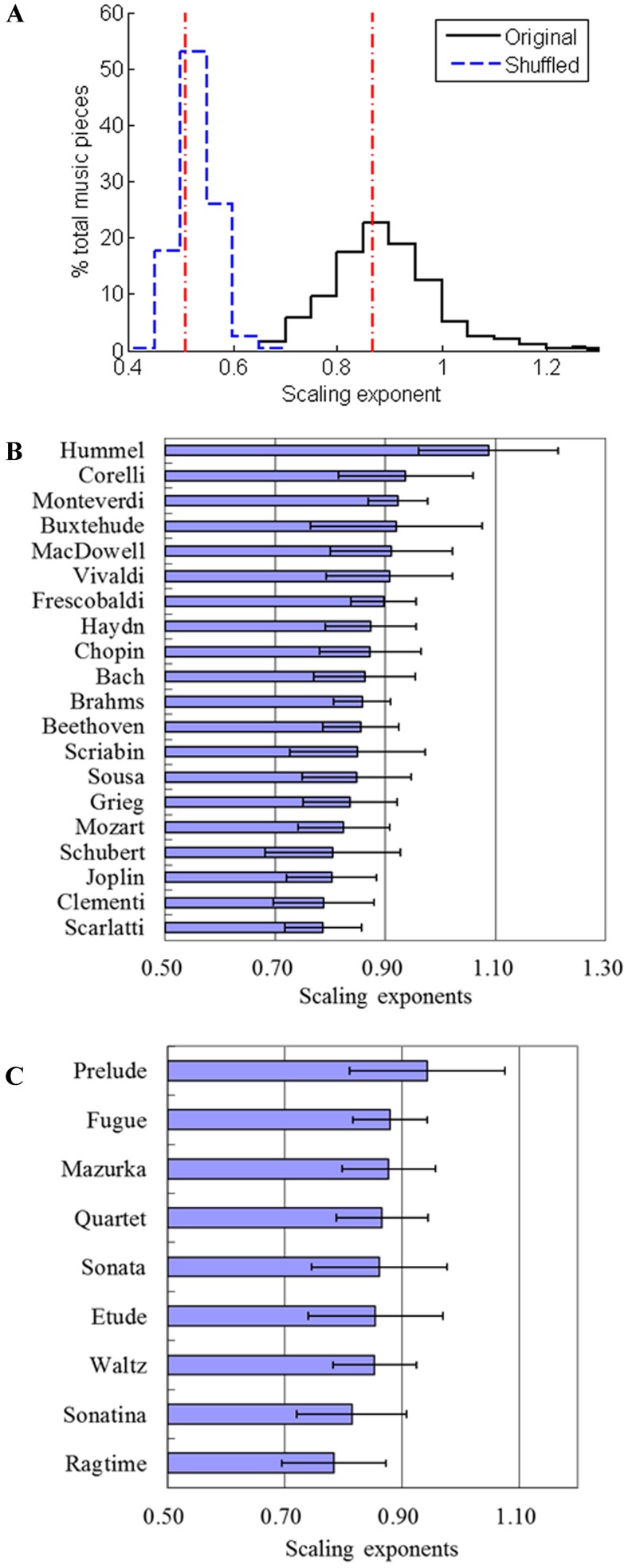
The scaling exponents for all the music movements across different composers and genres. (A) The scaling exponent of all the 1191 musical movements and the corresponding shuffled counterparts. (B) The scaling exponents for 20 composers. (C) The scaling exponents for 9 different genres.

The results of all the 1191 movements are shown in Fig 3. The average scaling exponent is 0.87, while the average exponent for the shuffled signals is 0.51 (Fig 3A). This finding demonstrates that across the analyzed compositions, the fluctuation of harmony is characterized by the 1/f power law. We found the average exponent for all the 20 composers was approximately 0.9 (Fig 3B, Table 1), indicating that the consonant intervals do indeed exhibit 1/f structure. The shuffled series showed an α value of approximately 0.5, i.e. akin white noise, which was significantly different from the origin (*P*<0.01, Table 1). This provides strong evidence for the 1/f characteristic of the consonance information. The composer Hummel (1778–1837) had the largest α value (1.1±0.1) (mean±s.d.). Beethoven (α = 0.86±0.07) (1770–1827), who lived at approximately the same period, had a significantly different distribution of exponents from Hummel (*P*<0.05). However, “the three Bs” in classical music history, i.e., Bach (α = 0.86±0.09) (1685–1750), Brahms (α = 0.86±0.05) (1833–1897) and Beethoven, had equivalent exponents, despite living in different eras. These results indicate that composers may have different characteristics despite living in the same era, and the similarity of the CR properties may not be correlated with the era of the composers. Fig 3C shows the results for 9 different genres and confirms that they all follow the scale-free law. The α values range from 0.78 to 0.94 and are significantly different from their respective shuffled counterparts (*P*<0.01, Table 2). The preludes had the largest α values, indicating the most consonant interval variety. The ragtime genre had the smallest α value among these genres, indicating that ragtime has a unique consonance fluctuation.

**Fig 4:**
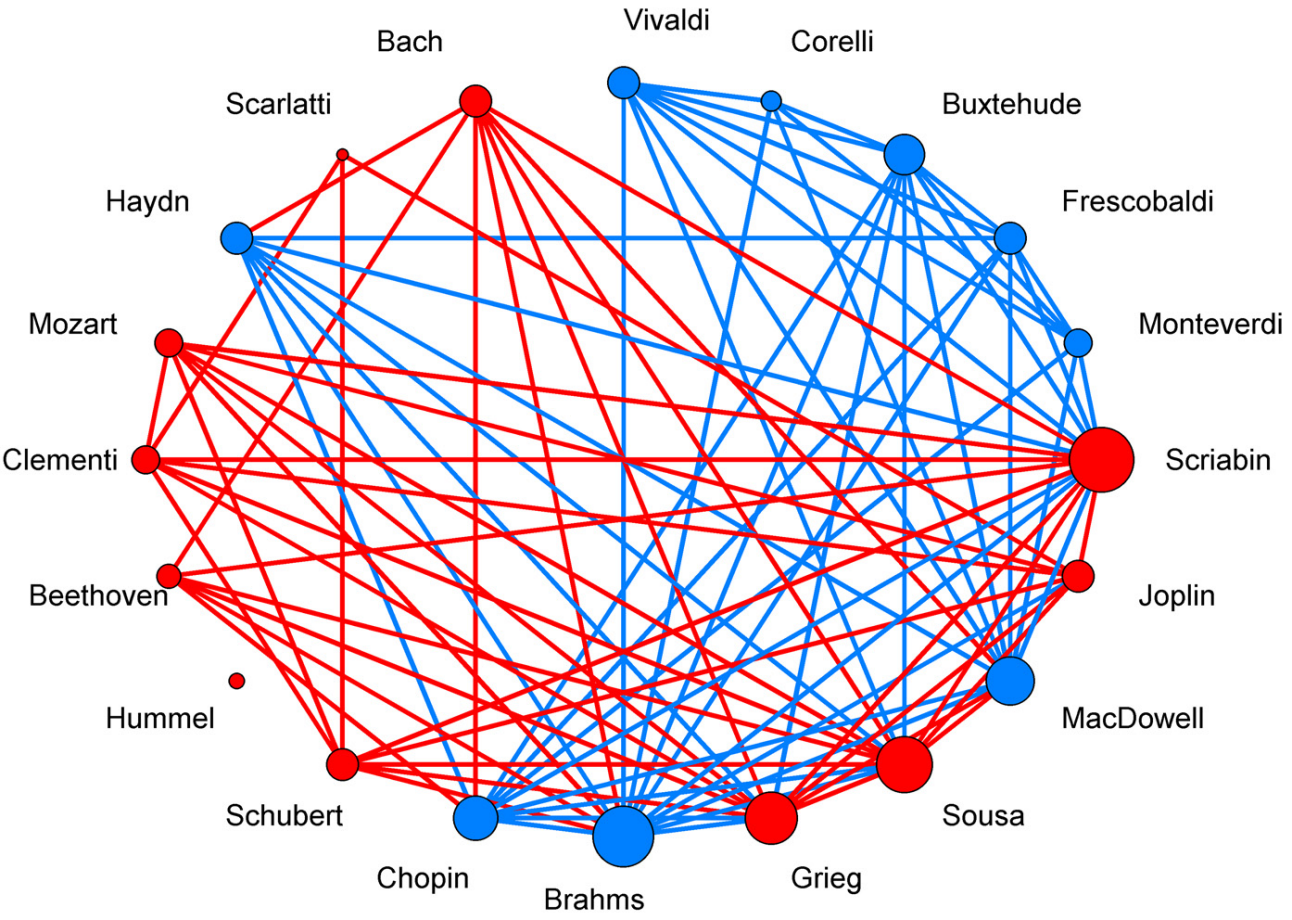
The network of the composers. The nodes corresponded to the composers, and the connections among the nodes are established according to the difference between the exponents of any two composers over all of their movements. When the difference is not significant (*P*>0.05) in t test, a line linking the two composers is assumed. The composers in the network were put into two groups (red and blue) according to the network modularity. The size of the circle indicates the degree (the number of lines).

To evaluate the inherited relationships among different musicians, a network diagram of composers was constructed based on their exponent values of CR series (Fig 4). This network analysis reveals that the composers who lived in later eras usually had greater exponent values than those who lived in earlier ones. The composer Hummel was completely isolated in this analysis, indicating that he was unique in his use of musical consonance. Indeed, some critics have suggested that Hummel’s music took a different direction from that of Beethoven by challenging the classical harmonic structures and stretch the sonata form [37]. The second-smallest degree belonged to Scarlatti; his use of the Phrygian mode and other tonal inflections was relatively alien to European music at the time. Many of Scarlatti’s dissonances and figurations were suggestive of the guitar [38]. Brahms had a large degree in the network, and it was known that he venerated Beethoven; some passages in his works were reminiscent of Beethoven’s style [39]. Brahms was also influenced by Mozart, Haydn and Bach [39]. These wide relationships demonstrate the broad links between Brahms and other composers. The composers Scriabin and Sousa lived in the 20th century, and thus had learned from many predecessors and had greater degrees. The only small degree in the Romantic era was that belonging to Joplin, who was famous for his unique musical style, ragtime. This result indicates that Joplin’s ragtime was novel in terms of dissonance fluctuation.

There were two groups in the composer network according to the network modularity analysis. This supports the notion of Bach as “the father of harmony” since composers before Bach were in one group whereas most after him were in his group (10 in Bach’s group, 4 in the other group). Even the composers after Bach who were not in his group were shown to be connected to him by the network analysis.

**Fig 5:**
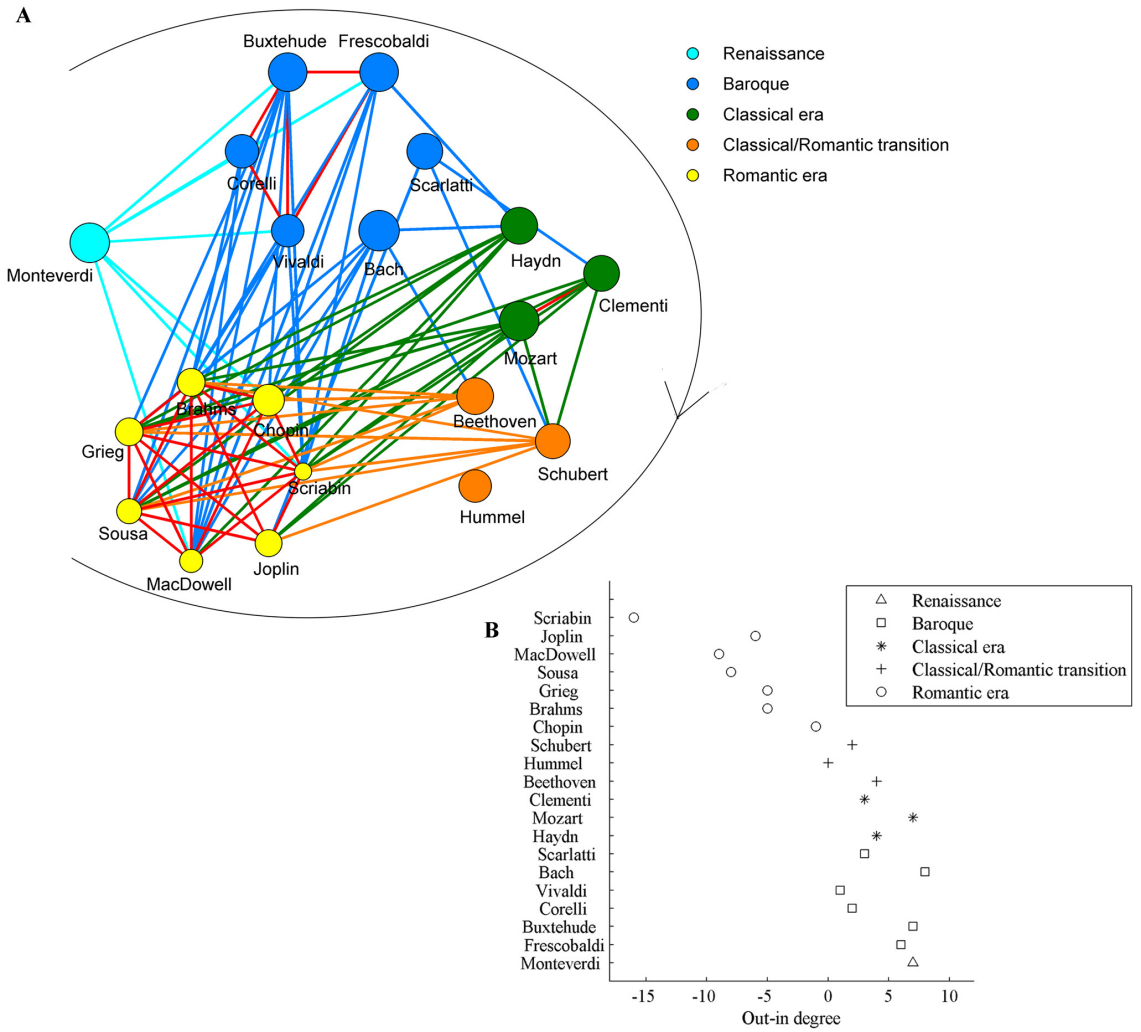
The network of composers in different eras. (A) The composers are arranged according to the eras they belonged to and different colors are used to identify the eras. The arrow around all the nodes represents the time. The size of the circle indicates the influence of the composer (out-in degree). Red lines are used for composers within era. (B) The influence (out-in degree) of the nodes in the composers’ network.

We further analyzed the relations of these composers with their eras. As shown in Fig 5A, we found that in the Baroque era, composers had connections other than with Bach and Scarlatti. In the classical era, only Mozart and Clementi had connections. The composers in the transition era had no connections whereas those in the Romantic era had strong connections among them. This indicates that the musicians in the Baroque and Romantic eras influenced each other. We also found that composers before and within the Baroque era affected almost all subsequent ones, expecially the Romantic era. The composers in the Classical and transition periods showed fewer connections with Baroque, but more connetions with Romantic ones. Thus Classical and transition era composers had styles very different from previous ones. Overall, Bach had the greatest influence, which lends credence to Bach’s title of the “original father of harmony” [40]. Mozart, Beethoven, Chopin and Brahms were among his most prominent admirers; they began writing in a more contrapuntal style after being exposed to Bach’s music. The second-most influential composer was Mozart (according to this analysis), who was clearly established as an important figure in music history and about whom Joseph Haydn wrote that “posterity will not see such a talent again in 100 years” [41]. Bach, Mozart and Beethoven had almost no connections with composers before them. On the other hand they had many connections with composers after them. We also defined a cultural heritage direction of the network edges according to their relative birth order, and then the out-in degree was obtained as an indicator of influence. A high positive value of the out-in degree may indicate that the composer had an important influence on the evolution of music, similar to the source of musical lineages, whereas a low or negative out-in degree means that the composer mainly inherited their style from older generations (Fig 5B). Together these findings indicate that Bach and Mozart were both the most influential in their own eras, as well as on the works of the composers in subsequent eras, thereby further confirming their reputation as key figures in the history of music. The analysis shows that after Bach and Mozart, Monteverdi, Frescobaldi and Buxtehude were the most influential and their works were also occasionally imitated by others.

**Fig 6:**
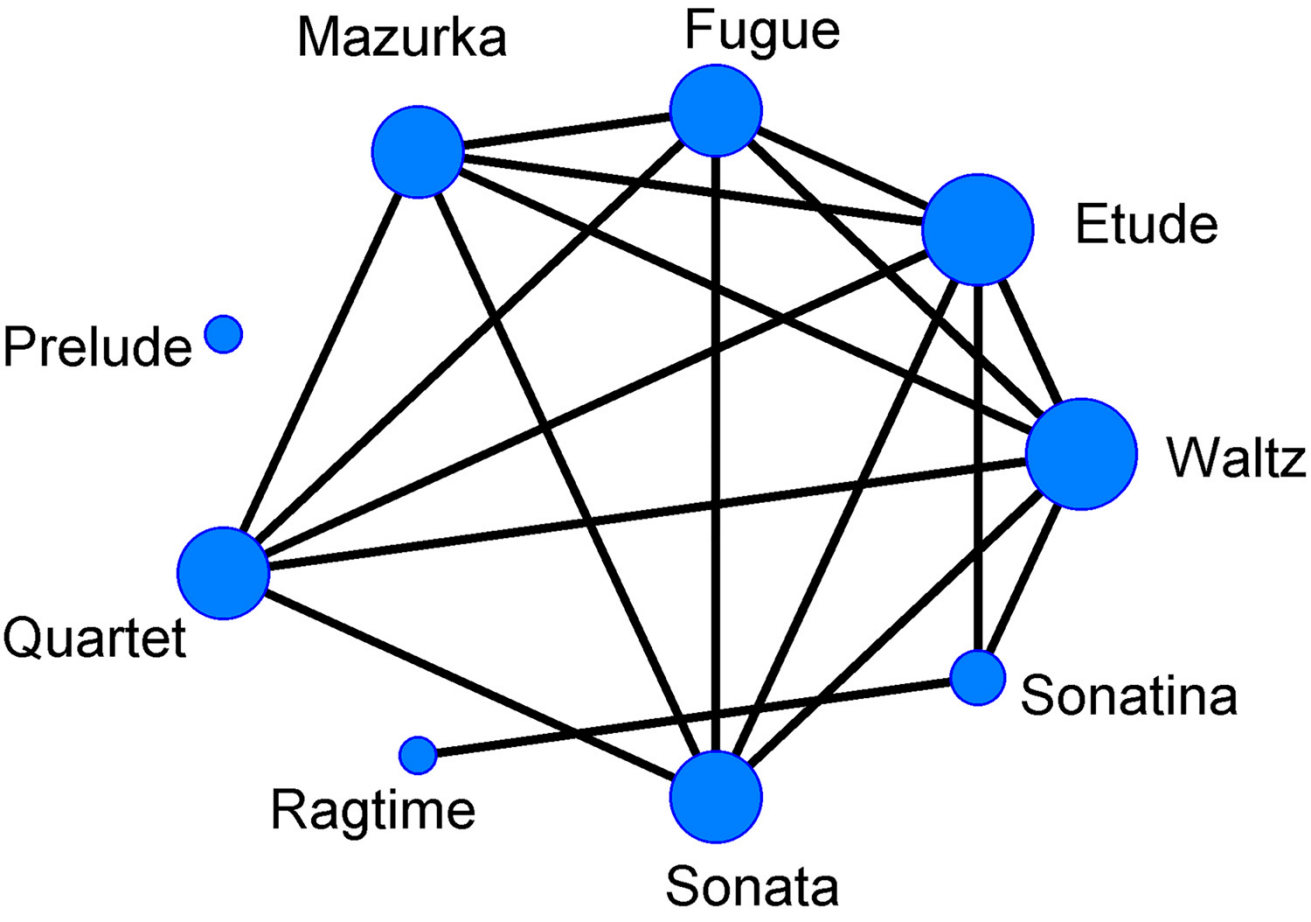
The network of genres. When the difference of any two genres is not significant (*P*>0.05) in a t test, a line between the two genres is assumed. The size of the nodes represents the degrees.

Similarly, we constructed a network of genres (Fig 6) which revealed that the waltz and the etude had the largest degrees and that the smallest ones corresponded to ragtime and prelude genres. The waltz is defined by its strong rhythmic pattern and has remained popular for hundreds of years [42], so it is not surprising that its harmony influences many other genres. Etudes are usually short and difficult to play; designed to provide practice material for perfecting specific technical skills [42] and include many varieties of consonance. Ragtime and prelude are somewhat specialised genres, and thus their degrees are unsurprisingly small. The broad relationships among all the studied genres occurred because they were widely adopted by various composers of different eras suggesting that some specific consonant interval might be at the heart of all of the various genres causing them to be widely linked. Indeed, current practice in musical analysis emphasises an important role for the consonant interval series in various genre studies and the role of harmony for genre classification [27].

## Discussion

In summary, we have demonstrated an interesting characteristic of the music harmony. Previous studies have demonstrated the scale-free properties of the pitch [15,18,43], rhythm [13], intensity [44], melody [15–17] and structure [45] of a large number of musical compositions. The current findings show for the first time that consonance fluctuation in music obeys the same law.

In fact, as comparisons, we also calculated the pitch and chord fluctuation for the above music movements (Table 1) in this work. The pitch series were extracted from the highest pitch at each time point. For example, in Fig 1C and 1B (71) would be chosen as the representative pitch. For the chord fluctuation analysis, the key of the movement was found first [46]; then the chord name and its stability rank. At last, the chord stability rank series were obtained (S1 Table).

The average DFA exponent of pitch was 0.9, which was larger than that of the consonance interval. For composers, Grieg, Bach, Frescobaldi and Corelli, the values were very close to 1.0. For genres, the scaling exponents of pitch were all larger than that of consonance interval, and prelude and fugue were the largest and second largest values, the trend was the same as the consonance fluctuation (S1 Fig). These results support that pitch fluctuation does obey the 1/f structure [18].

The average DFA exponent of chord fluctuation was 0.87, and the composer Hummel had the largest α value (1.0±0.1). Beethoven (α = 0.85±0.07) had a significantly different distribution of exponents from Hummel (*P*<0.05). “The three Bs” in classical music history, i.e., Bach (α = 0.87±0.09), Brahms (α = 0.88±0.05) and Beethoven, had approximate equivalent exponents, despite living in different eras. For different genres, preludes had the largest α values, while ragtime had the smallest α value among these genres (S2 Fig). Interestingly, these relative relations are similar to those of consonance intervals.

According to the consonance fluctuation, composers Bach, Mozart et al. displayed their special characters in musical history. However, according to pitch fluctuation, the influence was based on the eras (S3 Fig). Composers in early eras had high out-in degree, while composers in late eras had low out-in degree. This tendency existed in chord fluctuation, too, except with Monteverdi and Mozart (S4 Fig). However, Monteverdi showed lower influence than composers of Baroque era, indicating that composers in Baroque had high influence than in Renaissance for chord utilizing. Mozart had quite high influence suggested that his feature of chord using affected other composers. The influence of Bach was not a highlight in pitch and chord. The reason may be that Bach’s famous counterpoint works was not expressed in pitch and chord fluctuation, but showed in consonance. Therefore, as “the father of harmony”, Bach actually played the most important role in the counterpoint works and established a new way for musical harmony using through interval consonance.

The scale-free distribution of musical properties is thought to be related to their aesthetic quality [14,15]. An optimal balance of predictability and surprise may cause the pleasing feelings in music appreciation [47]. When music is played, listeners expect the next note, not only for its pitch, duration, but also for the harmony. Compared to these basic music elements such as pitch, duration, harmony is more complicated and considered to be a distinction central to Western music [48]. The harmony reflects cultural customs [49], for there are specific styles without using harmony, like Chinese folk music. However, there are different opinions concerning whether the perception of specific harmony patterns is innate or not. Whatever the preference for consonant intervals is underlain by familiarity [50], or is a production of neurons' firing action potentials [51] and the brainstem temporal coding [52], our findings in this study provides some evidences that music consonance fluctuations obey the 1/f law across centuries, especially with the most famous composers in history. That may be a new way to investigate the relation between human and music harmony.

Actually, human perception is known to focus on scale-free signals in the environment [53,54], and physiological signals follow the same law, as evidenced by patterns of brain electrical activity [54–56]. The consonance plays an important role in music perception. Since the human nerves are sensitive to 1/f noise, the harmony pattern of that structure exists when the composers wrote their works. The products of human creativity such as music, painting are also frequently inspired by our experience of the natural world. Although individual differences may enrich the variety of creative expression and shape its evolution, the relatively stable influence of the scale-free framework provides a platform ensuring fundamental relationship between the artistic works of different individuals. This also effectively creates a defining feature that characterises, encompasses and sets the boundaries for all forms of human art. It also resonates strongly with the Chinese traditional idea of the “oneness of man and nature”.

The consonance in music can cause pleasant feeling while dissonance may cause unpleasant one [48], and they elicit different EEG gamma activity [57]. The brain networks are different when the musicians perform music in a mechanical manner or a more emotionally rich manner [58]. And the harmony progressions may enhance the emotion expression [24]. So the harmony features are used for emotion recognition [59]. Therefore, the 1/f distribution may be a bridge between music harmony fluctuation and the emotion. The method developed in our study is likely to be useful in musical analysis, emotion recognition etc. Although the consonance fluctuation is not the whole story of harmony, it does provide some meaningful information about harmony. Additional features of harmony will be worthwhile to analyze in the future.

We have demonstrated an intrinsic heritage relationship based on patterns of scale-free harmony across a representative range of musicians from different eras spanning four centuries. Is this mathematically established relationship reasonable? In a recent artificial music experiment, consonance was also confirmed to be an important factor in determining musical evolution [60]. In Trehub’s model for the evolution of music, humans are hypothesized to have adopted music to help soothe infants or focus their attention [61] by using a variety of consonant intervals to induce calm or tense feelings [62]. Tension created by music is associated with the power of the chord progression and this is influential in music development. Our results suggest that relationships among the studied composers based on consonant interval may provide a novel and quantitative way of understanding music throughout history and may present a useful method for studying its structure and roots.

## Supporting Information

S1 Fig The scaling exponents of the all music movements across different composers and genres based on pitch series. (A) The scaling exponents for 20 composers. (B) The scaling exponents for 9 different genres.
(TIF)

S2 Fig The scaling exponents of the all music movements across different composers and genres based on chord series. (A) The scaling exponents for 20 composers. (B) The scaling exponents for 9 different genres.
(TIF)

S3 Fig The influence (out-in degree) of the nodes in the composers’ network based on pitch series. (TIF)

S4 Fig The influence (out-in degree) of the nodes in the composers’ network based on chord series. (TIF)

S1 File The MIDI files of all the composers and genres. (ZIP)

S1 Table The chord name and stability rank of major C and minor a. The number in the table means the scale degree of a chord. Ⅰis tonic, Ⅴis dominant and Ⅳ is subdominant chord. They are the primary harmonies in music. In the key of C major, chordⅠis named C, which consists of note C, E, G. When we use integer 0–11 to represent the notes in an octave, (C, E, G) are (0, 4, 7). The stability rank of chord is according to the number, and it is related to the consonance rank (Table 1). For example, the interval between root note of Ⅴ and Ⅰis perfect fifth, so the stability rank of Ⅴ is 2, the same as the consonance rank of interval “perfect fifth”. The stability rank of a chord consisted of more than three notes is 8.
(DOCX)
