# Supplementary figures and images for: Bach Is the Father of Harmony: Revealed by a 1/f Fluctuation Analysis across Musical Genres
Source: PLoS One. 2015 Nov 6;10(11):e0142431. doi: 10.1371/journal.pone.0142431 (PMC4636347; doi:10.1371/journal.pone.0142431)

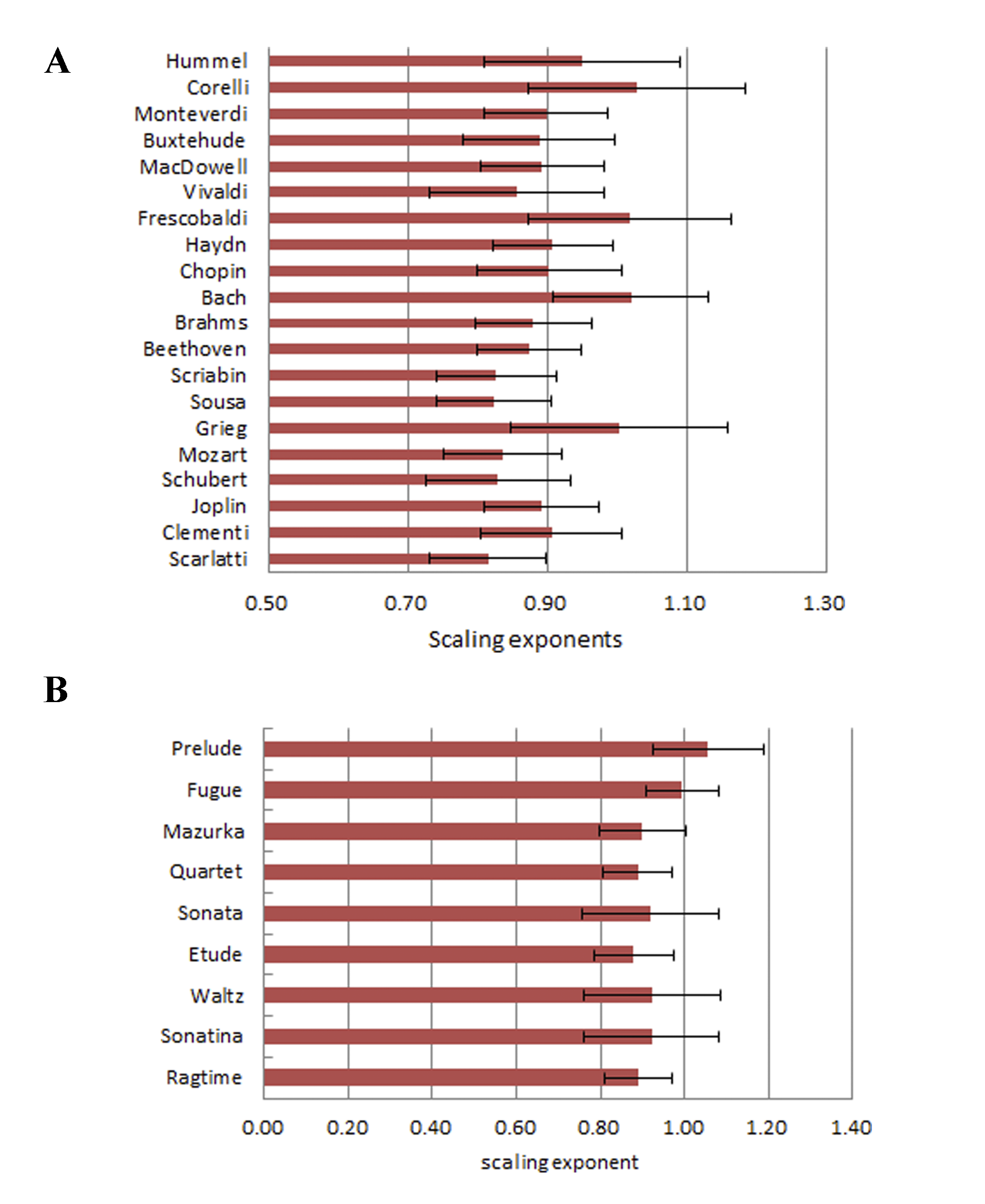

Supplement: S1 Fig — (A) The scaling exponents for 20 composers. (B) The scaling exponents for 9 different genres. (TIF) [file pone.0142431.s001.tif]

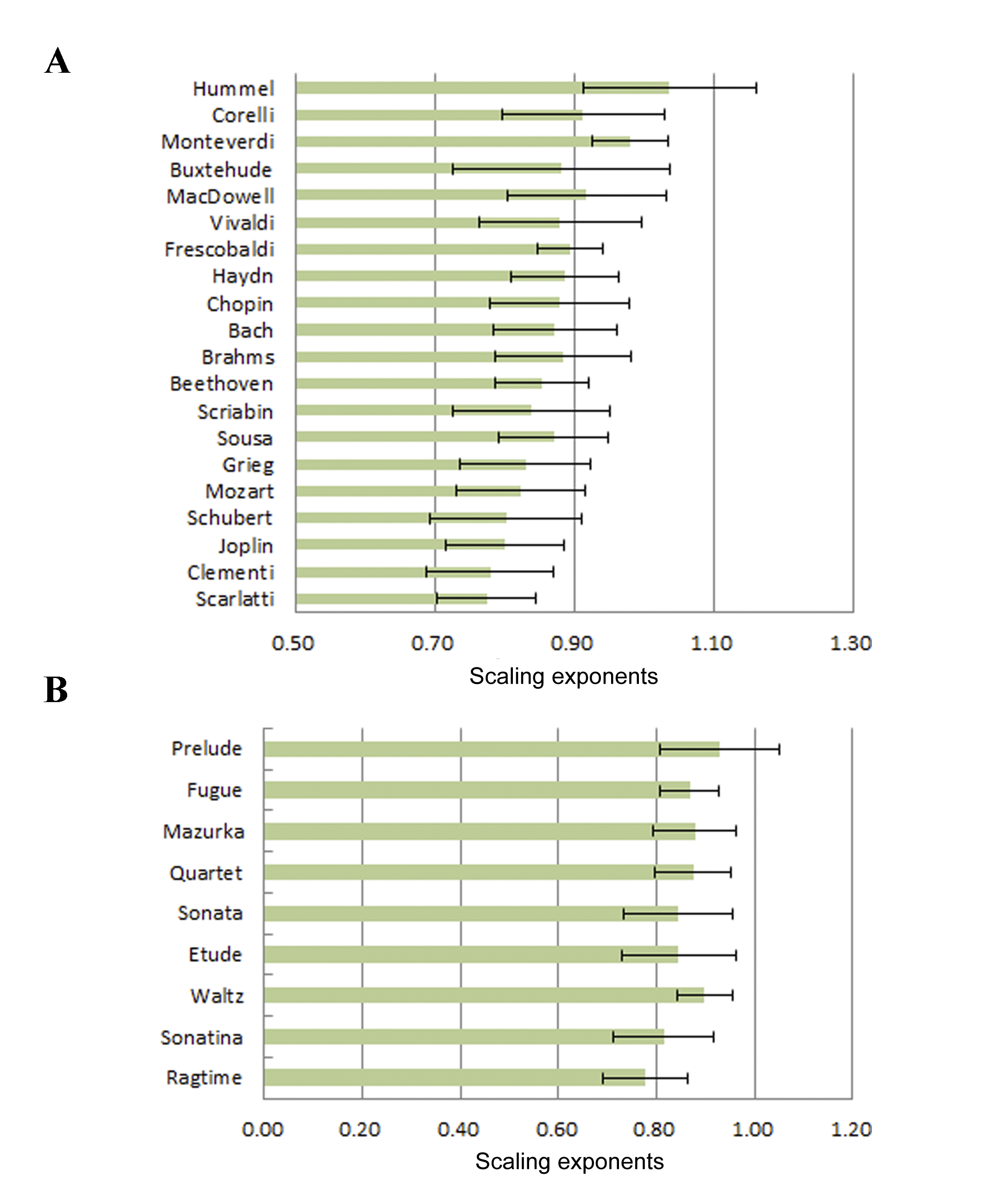

Supplement: S2 Fig — (A) The scaling exponents for 20 composers. (B) The scaling exponents for 9 different genres. (TIF) [file pone.0142431.s002.tif]

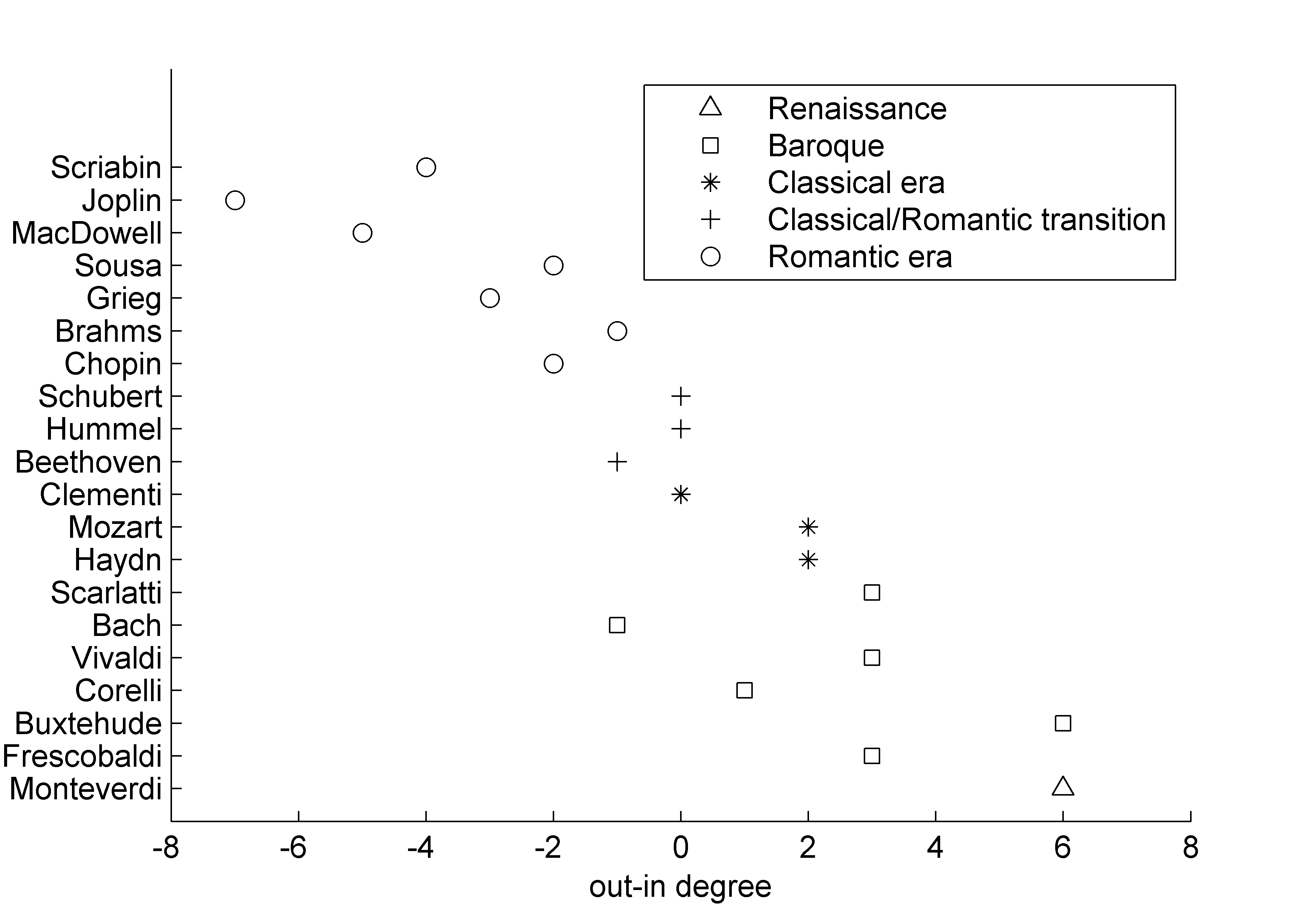

Supplement: S3 Fig — (TIF) [file pone.0142431.s003.tif]

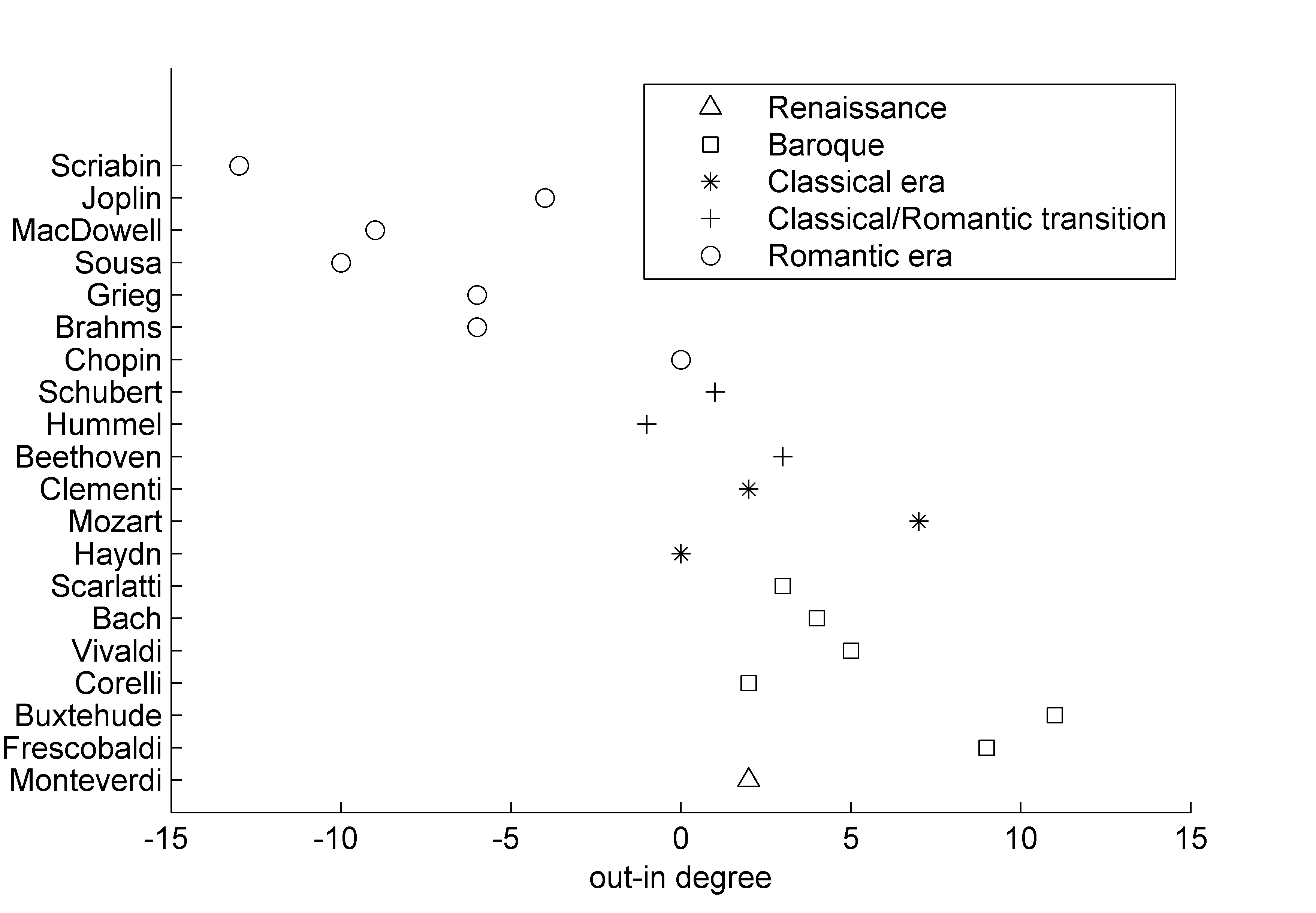

Supplement: S4 Fig — (TIF) [file pone.0142431.s004.tif]
