# Supplementary material for: Bach Is the Father of Harmony: Revealed by a 1/f Fluctuation Analysis across Musical Genres
Source: PLoS One. 2015 Nov 6;10(11):e0142431. doi: 10.1371/journal.pone.0142431 (PMC4636347; doi:10.1371/journal.pone.0142431)
Supplement: S1 Table — The number in the table means the scale degree of a chord. Ⅰis tonic, Ⅴis dominant and Ⅳ is subdominant chord. They are the primary harmonies in music. In the key of C major, chordⅠis named C, which consists of note C, E, G. When we use integer 0–11 to represent the notes in an octave, (C, E, G) are (0, 4, 7). The stability rank of chord is according to the number, and it is related to the consonance rank (Table 1). For example, the interval between root note of Ⅴ and Ⅰis perfect fifth, so the stability rank of Ⅴ is 2, the same as the consonance rank of interval “perfect fifth”. The stability rank of a chord consisted of more than three notes is 8. (DOCX) [file pone.0142431.s006.docx]

**S1 Table. The chord name and stability rank of major C and minor a.**

|  | C major | | a minor | |  |
| --- | --- | --- | --- | --- | --- |
| Number | Name | Chord note | Name | Chord note | Stability rank |
| Ⅰ | C | (0, 4, 7) | Am | (0, 4, 9) | 1 |
| Ⅱ | Dm | (2, 5, 9) | Bdim | (2, 5, 11) | 6 |
| Ⅲ | Em | (4, 7, 11) | C | (0, 4, 7) | 4 |
| Ⅳ | F | (0, 5, 9) | Dm | (2, 5, 9) | 3 |
| Ⅴ | G | (2, 7, 11) | Em | (4, 7, 11) | 2 |
| Ⅵ | Am | (0, 4, 9) | F | (0, 5, 9) | 5 |
| Ⅶ | Bdim | (2, 5, 11) | G | (2, 7, 11) | 7 |
